# Lithium Promotes Neuronal Repair and Ameliorates Depression-Like Behavior following Trimethyltin-Induced Neuronal Loss in the Dentate Gyrus

**DOI:** 10.1371/journal.pone.0087953

**Published:** 2014-02-04

**Authors:** Masanori Yoneyama, Tatsuo Shiba, Shigeru Hasebe, Kasumi Umeda, Taro Yamaguchi, Kiyokazu Ogita

**Affiliations:** Department of Pharmacology, Setsunan University Faculty of Pharmaceutical Sciences, Hirakata, Osaka, Japan; University of Victoria, Canada

## Abstract

Lithium, a mood stabilizer, is known to ameliorate the stress-induced decrease in hippocampal neurogenesis seen in animal models of stress-related disorders. However, it is unclear whether lithium has beneficial effect on neuronal repair following neuronal damage in neuronal degenerative diseases. Here, we evaluated the effect of *in vivo* treatment with lithium on the hippocampal neuronal repair in a mouse model of trimethyltin (TMT)-induced neuronal loss/self-repair in the hippocampal dentate gyrus (such mice referred to as “impaired animals”) [Ogita et al. (2005) J Neurosci Res 82: 609–621]. The impaired animals had a dramatically increased number of 5-bromo-2′-deoxyuridine (BrdU)-incorporating cells in their dentate gyrus at the initial time window (days 3 to 5 post-TMT treatment) of the self-repair stage. A single treatment with lithium produced no significant change in the number of BrdU-incorporating cells in the dentate granule cell layer and subgranular zone on day 3 post-TMT treatment. On day 5 post-TMT treatment, however, BrdU-incorporating cells were significantly increased in number by lithium treatment for 3 days. Most interestingly, chronic treatment (15 days) with lithium increased the number of BrdU-incorporating cells positive for NeuN or doublecortin in the dentate granule cell layer of the impaired animals, but not in that of naïve animals. The results of a forced swimming test revealed that the chronic treatment with lithium improved the depression-like behavior seen in the impaired animals. Taken together, our data suggest that lithium had a beneficial effect on neuronal repair following neuronal loss in the dentate gyrus through promoted proliferation and survival/neuronal differentiation of neural stem/progenitor cells in the subgranular zone.

## Introduction

The concept that the adult mammalian brain contains populations of endogenous neural stem/progenitor cells (NPCs) has been widely accepted [1], [2]. Adult neurogenesis occurs in 2 particular regions in the brain, i.e., the subventricular zone of the lateral ventricles and the subgranular zone (SGZ) of the dentate gyrus in the hippocampus [3], [4]. For the production of new neurons, NSCs go through a process of proliferation, migration, differentiation, survival, and integration, thereby becoming productive members of the existing circuitry in the brain. Even under normal physiological conditions in the adult, NSCs predominantly produce neurons including interneurons in the olfactory bulb in the case of NPCs derived from the subventricular zone and neuronal cells in the dentate gyrus in the case of NPCs derived from the SGZ. These NPCs have the ability to respond to brain damage by producing neural cells including neurons, astrocytes, and oligodendrocytes [5]. Through enhancement of neural repair processes, i.e., proliferation, migration, differentiation, and survival, NPCs have the ability to replace cells damaged/lost following neural injury with new neuronal and glial cells. Indeed, brain ischemia enhances neurogenesis in both the subventricular zone and the SGZ [6]–[8]. Ischemia-induced cell proliferation and neurogenesis are considered as being a compensatory mechanism in response to neuronal loss. Therefore, treatment that enhances the neuronal repair process has been speculated to be a beneficial therapy for neuronal injury or neurodegenerative disorders.

The organotin trimethyltin chloride (TMT) is a neurotoxin that produces neuronal degeneration in both human and rodent central nervous systems [9]. A single systemic treatment of mice with TMT causes neuronal loss in restricted brain regions including the dentate gyrus, olfactory bulb, anterior olfactory nucleus, and frontal cerebral cortex [10]–[13]. Our previous studies using mice also demonstrated that TMT treatment markedly produces enhanced neurogenesis in the dentate gyrus and olfactory bulb through proliferation of NPCs in each of these brain regions [14]–[16]. These previous findings indicate that the TMT-treated mouse is a very attractive model for studies on neuronal self-repair (regeneration) following neuronal loss in the dentate gyrus.

The mood stabilizer lithium is used for treatment of stress-related disorders, and increases neurogenesis in the adult hippocampus [17]–[19]. These studies suggest that the therapeutic action of lithium in stress-related disorders might be due to enhanced neurogenesis in the hippocampus. Indeed, it is reported that glucocorticoid suppresses neurogenesis without causing neuronal damage in the hippocampus and that this suppression is ameliorated by lithium [20]. However, the effect of lithium on neurogenesis following crucial neuronal loss in the hippocampal dentate gyrus has been not evaluated. Elucidating how lithium regulates neurogenesis following hippocampal neuronal loss might provide a better understanding leading to the development of new therapeutic targets for neurodegenerative disorders. Therefore, the aim of the present study was to elucidate the effect of lithium on neuronal regeneration following neuronal loss in the dentate gyrus in the TMT-treated mouse, which is a model for neuronal loss/self-repair in the dentate gyrus.

## Materials and Methods

### Materials

Anti-goat IgG antibody conjugated to fluorescein isothiocyanate was purchased from Jackson ImmunoResearch Laboratories (West Grove, PA, USA). Rabbit polyclonal antibodies against ionized calcium-binding adapter molecule 1 (Iba1; Wako Pure Chemical Industries, Ltd., Osaka, Japan) and β-catenin (Sigma-Aldrich Co., St. Louis, MO, USA), goat polyclonal antibody against doublecortin (DCX; Santa Cruz Biotecchnology, Santa Cruz, CA), rat monoclonal antibody against 5-bromo-2′-deoxyuridine (BrdU; Abcam, Ltd., Cambridge, MA, UK), and mouse monoclonal antibodies specific for glial fibrillary acidic protein (GFAP; Sigma-Aldrich Co., St. Louis, MO, USA), nestin (Millipore Co., Boston, MA, USA), and neuronal nuclear antigen (NeuN; Chemicon International, Temecula, CA, USA) were used as primary antibodies. Alexa-Fluor 594-conjugated anti-rat IgG (H+L) antibody, Alexa-Fluor 488-conjugated anti-rabbit IgG (H+L) antibody, and Alexa-Fluor 488-conjugated anti-mouse IgG (H+L) antibody were obtained from Molecular Probes (Eugene, OR, USA). Lithium carbonate and TMT were supplied by Wako Pure Chemical Industries, Ltd. (Osaka, Japan). All other chemicals used were of the highest purity commercially available.

### Drug Administration and Experimental Schedules

The protocol used here met the guidelines of The Japanese Society for Pharmacology and was approved by the Committee for Ethical Use of Experimental Animals at Setsunan University. All efforts were made to minimize animal suffering, to reduce the number of animals used, and to utilize alternatives to *in vivo* techniques. Adult male Std-ddY mice weighing 26–28 g were housed in metallic breeding cages in a lighted room and given free access to food and water for at least 4 days before use. The mice were intraperitoneally injected with TMT (2.9 mg/kg) dissolved in phosphate-buffered saline (PBS) for preparing the mouse model of neuronal loss/self-repair in the hippocampal dentate gyrus (hereafter collectively referred to as “impaired animals”). Other mice were given PBS of the same volume as that of the TMT solution and hereafter collectively referred to as “naïve animals.” Lithium carbonate (100 mg/kg) was dissolved in PBS and intraperitoneally injected into the animals once a day for the desired number of days, starting on day 2 post-TMT treatment. To label mitotic cells, we gave mice a single series of 2 consecutive injections of BrdU (50 mg/kg, i.p., dissolved in PBS) at a 12-h interval on day 2 post-TMT treatment. These animals were then returned to their home cages until the time of decapitation.

**Figure 1:**
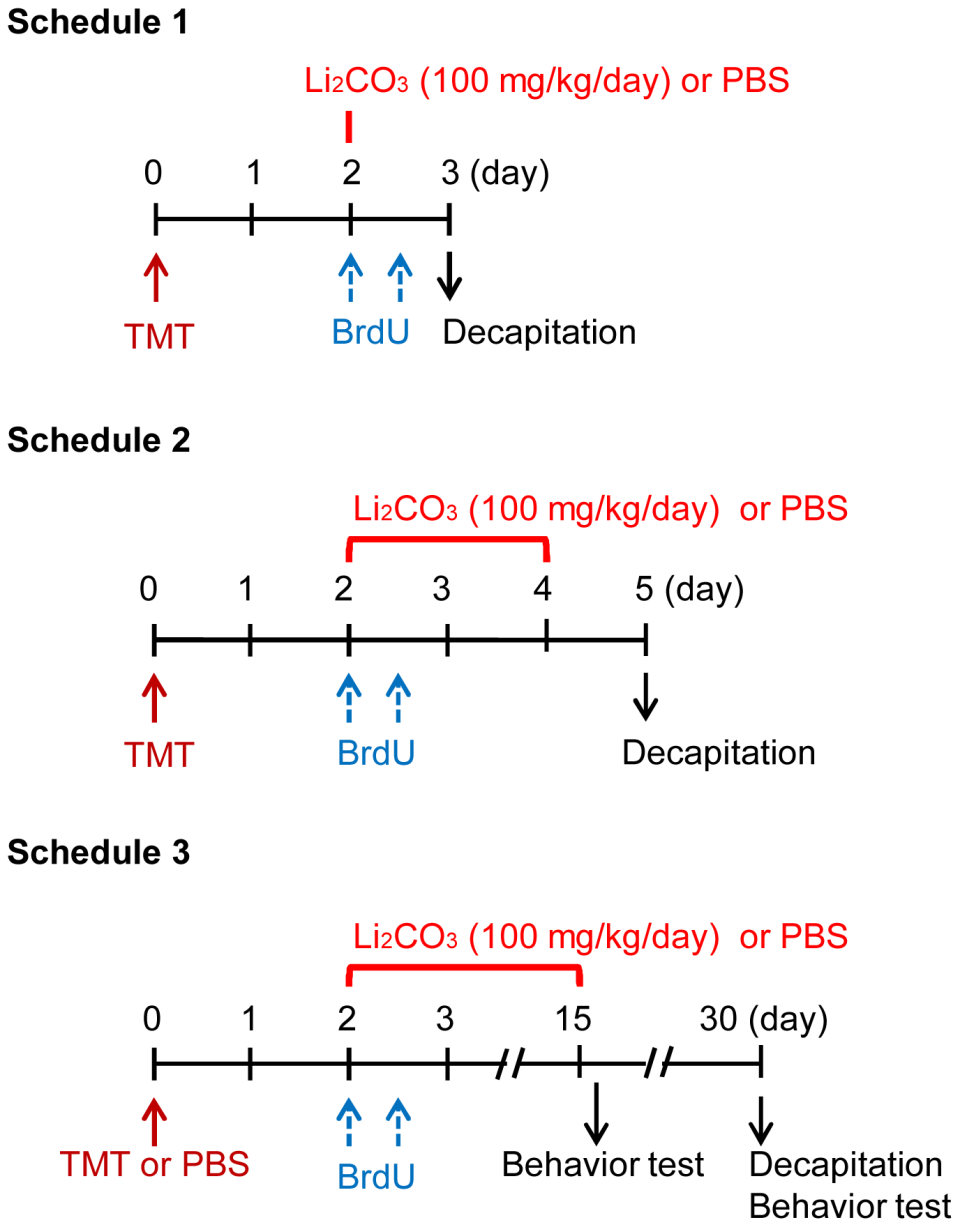
Experimental schedules. In “Schedule 1, 2, and 3,” animals were given TMT (2.9 mg/kg, i.p.), and then received 2 consecutive injections of BrdU (50 mg/kg, i.p.) with a 12-h interval between them on day 2 post-TMT treatment for labeling mitotic cells in the dentate gyrus. To examine the effect of acute treatment with lithium carbonate on the proliferation of neural progenitor cells at the initial time window following neuronal loss in the dentate gyrus of the impaired animals, we carried out experiments under the conditions of “Schedule 1 or 2.” To examine the effect of chronic treatment with lithium carbonate on survival and differentiation of the newly-generated cells in the dentate gyrus of the impaired animals, we carried out experiments under the conditions of “Schedule 3.”

We divided the animals into 4 different groups for the experiments, i.e., PBS-treated naïve animal (naïve/PBS), lithium-treated naïve animal (naïve/Li), PBS-treated impaired animal (impaired/PBS), and lithium-treated impaired animal (impaired/Li). To examine the effect of acute and chronic treatments with lithium on the proliferation, survival, and differentiation of neural progenitor cells generated following TMT-induced neuronal loss in the dentate gyrus, we carried out experiments under 3 different schedules, i.e., “Schedule 1,” in which the animals were given either lithium or PBS on day 2 post-treatment with TMT and then decapitated 1 day later; “Schedule 2,” in which the animals were given either lithium or PBS daily on days 2 to 4 post-treatment with TMT and then decapitated 1 day later; and “Schedule 3,” in which the animals were given either lithium or PBS daily on days 2 to 15 post-treatment with PBS or TMT and then decapitated on day 30 post-treatment with PBS or TMT (Figure 1). In the case of Schedule 3, a forced swimming test was carried out on days 16 and 30 post-treatment with PBS or TMT.

### Preparation of Hippocampal Slices

Mice were anesthetized with chloral hydrate (500 mg/kg, i.p.) and perfused via the heart with PBS, followed by 4% (wt/vol) paraformaldehyde in 0.1 M sodium phosphate buffer (pH 7.4). The brains were quickly removed and further fixed with the same fixative solution at 4°C overnight. Post-fixed brains were embedded in paraffin, cut with a microtome into 7 sagittal sections of 3- to 5-µM thickness at 100-µm intervals in the range from 0.9 to 1.6 mm relative to lateral according to the atlas of Franklin and Paxinos [21] and placed on Matsunami-adhesive silane-coated glass slides (Matsunami Glass Ind., Kyoto). The paraffin-embedded brain sections were then deparaffinized with xylene, rehydrated by immersion in ethanol of graded decreasing concentrations of 100% (vol/vol) to 50% (vol/vol), and finally washed with water. Sections so obtained were subjected to the immunohistchemical procedures described below.

### Immunostaining

For double labeling of BrdU and each of NeuN, GFAP or Iba1, the sections in 10 mM sodium citrate buffer (pH 7.0) were first heated for 10 min in a microwave oven. After having been washed with TBST, they were blocked with 5% normal goat serum for 1 h at room temperature, and then incubated with the primary antibody against BrdU (3 µg/mL) and that against each of nestin (1 µg/mL), NeuN (3 µg/mL), GFAP (1∶600), Iba1 (1 µg/mL) or β-catenin (1∶2000) at 4°C overnight. After having been washed with TBST, they were next reacted with secondary antibodies (5 µg/mL Alexa Fluor 594-conjugated anti-rat IgG for BrdU; 5 µg/mL Alexa Fluor 488-conjugated anti-mouse IgG for nestin, NeuN, and GFAP; and 4 µg/mL Alexa Fluor 488-conjugated anti-rabbit IgG for Iba1) for 2 h at room temperature.

For double labeling using antibodies against BrdU and DCX, sections were first heated in the microwave oven in 10 mM sodium citrate buffer (pH 7.0) for 10 min. After having been washed with TBST, they were blocked with 5% normal horse serum for 1 h at room temperature, and then incubated with the primary antibodies against BrdU (3 µg/mL) and DCX (0.6 µg/mL) at 4°C overnight. After having been washed again with TBST, they were then reacted with fluorescein isothiocyanate-conjugated anti-goat IgG as the secondary antibody for DCX at room temperature for 2 h. After another wash with TBST, the sections were subsequently blocked with 5% normal goat serum for 20 min at room temperature and subsequently incubated with 5 µg/mL Alexa Fluor 594-conjugated anti-rat IgG for BrdU at room temperature for 2 h.

Double-stained sections were viewed with a BX41 microscope (Olympus, Tokyo, Japan) equipped with a DS-Ri1 camera (Nikon, Tokyo, Japan), and the number of highly labeled cells was counted by microscopic observation. To obtain the number of total positive cells per each animal, the 7 sagittal sections prepared from the brain of each animal were used for immunostaining and counting positive cells. X-positive cells, where X refers to a given antigen, were reported as X(+) cells.

### Behavioral Observations

For the forced swimming test, mice were forced to swim individually in a TPX beaker (18×26 cm; SANPLATEC) containing fresh water of 18-cm height and maintained at 25°C. After an initial period of vigorous activity, each animal assumed a typical immobile posture. A mouse was considered to be immobile when it remained floating in the water without struggling, making only the minimum movements of its limbs necessary to keep its head above water. The total duration of immobility was recorded during the 5-min test. The change in immobility duration was studied after treatment of individual animals with the drugs.

Locomotor activity was measured by using a digital counter system with an infrared sensor (Muromachi Kikai, Tokyo, Japan). Each mouse was placed individually in a black plastic cage (25-cm width×40-cm length×30-cm height), and the locomotor activity was measured for 30 min. All tests were carried out in a room illuminated by a 40-W white light suspended 2 m above the apparatus.

### Data Analysis

All data were expressed as the mean ± S.E.M., and the statistical significance was determined by use of the two-tailed Student *t*-test, one-way ANOVA with Bonferroni/Dunnett *post hoc* test or two-way repeated measures ANOVA.

## Results

### Effect of Acute Treatment with Lithium on Generation of BrdU(+) Cells following Neuronal Loss in the Dentate Gyrus

Our previous report indicated that the acute systemic treatment with TMT produces a marked neuronal loss in the dentate granule cell layer on day 2 post-treatment as well as cognitive impairment in mice [14]. Following the TMT-induced neuronal loss in the dentate gyrus, a marked increase in the number of BrdU-incorporating cells and of cells positive for nestin, NeuroD or DCX, which are neurogenesis-related markers, is seen in the dentate gyrus. Using this model of neuronal loss/self-repair in the dentate gyrus, we assessed the effect of lithium on neuronal regeneration following this neuronal loss.

**Figure 2:**
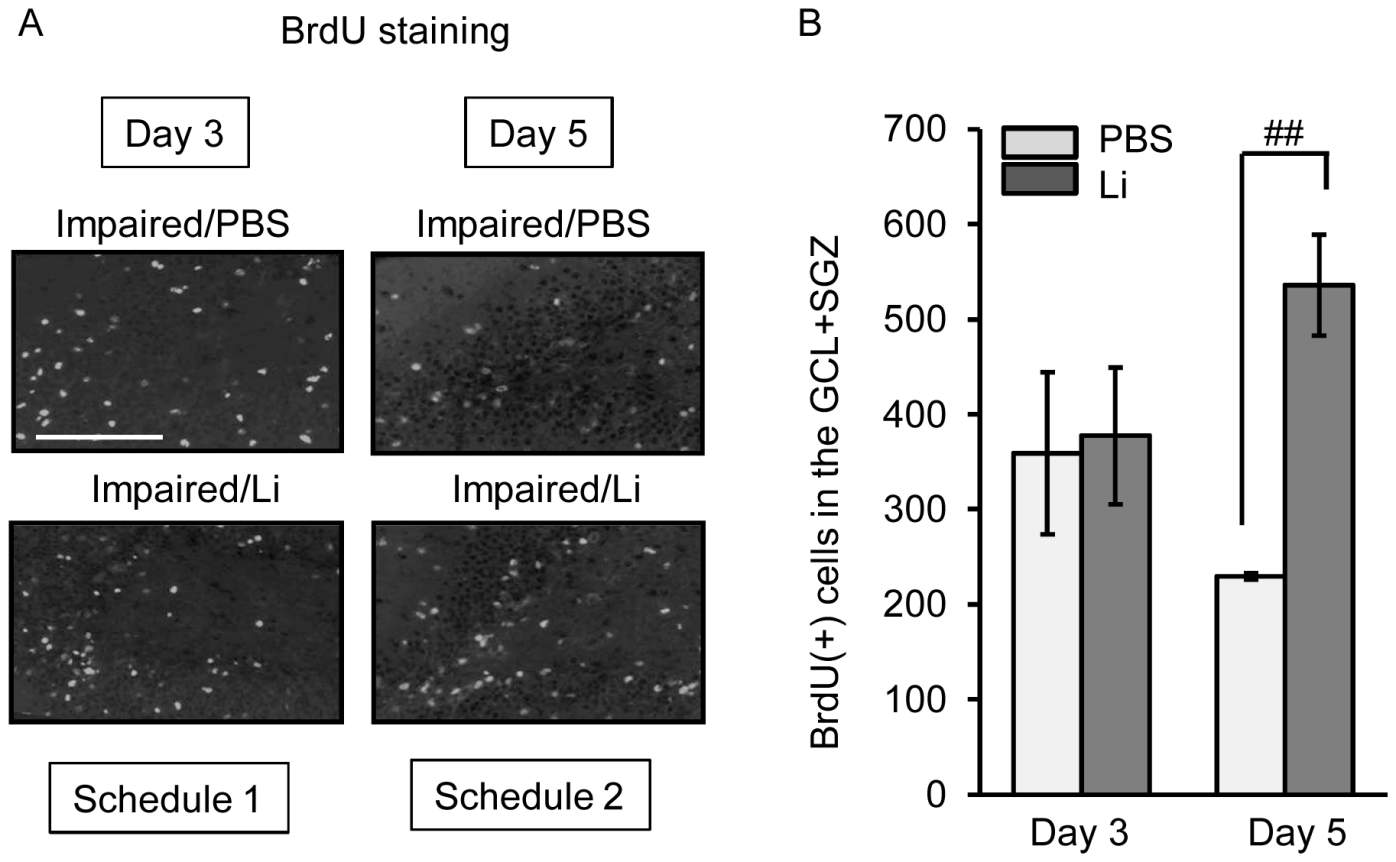
Effect of lithium (Li) on BrdU incorporation following neuronal loss. Animals were given either lithium carbonate (100 mg/kg, i.p.) or PBS alone with BrdU on day 2 post-treatment with TMT, and then decapitated on day 3 (Schedule 1). For Schedule 2, animals were given once a day either lithium carbonate (100 mg/kg, i.p.) or PBS on days 3 and 4, and then decapitated on day 5 post-TMT treatment. The sagittal hippocampal sections were then stained with anti-BrdU antibody. (**a**) Fluorescence micrographs show BrdU(+) cells in the dentate gyrus of the 2 groups (impaired/PBS, impaired/Li) on days 3 and 5 post-TMT treatment. Scale bar = 100 µm (**b**) The graph denotes the number of BrdU(+) cells in the GCL+SGZ of each group. Values are expressed as the mean ± S.E., calculated from 5 animals. ^##^ *P*<0.01, significant difference between the values obtained for PBS and Li groups.

To assess the effect of the acute treatment with lithium on the generation of BrdU-incorporating cells in the dentate gyrus of the impaired animals, we gave mice lithium at the dose of 100 mg/kg and BrdU on day 2 or days 2 to 4 post-treatment with TMT (Figure 2). A large number of BrdU(+) cells was found in the whole dentate gyrus including the GCL+SGZ, molecular layer, and hilus, as previously reported [14]. Of these regions, the GCL+SGZ had the largest proportion of BrdU(+) cells in the impaired animals. The single treatment with lithium produced no significant change in the expression of BrdU(+) cells in this region. Compared with the single treatment with lithium on day 2 post-TMT treatment, treatment with lithium daily on days 2 to 4 post-TMT treatment significantly increased the number of BrdU(+) cells in the GCL+SGZ. The significant increase between days 3 and 5 post-TMT treatment was due to not only a decrease in the number in the PBS group but also an increase in the number in the lithium group.

**Figure 3:**
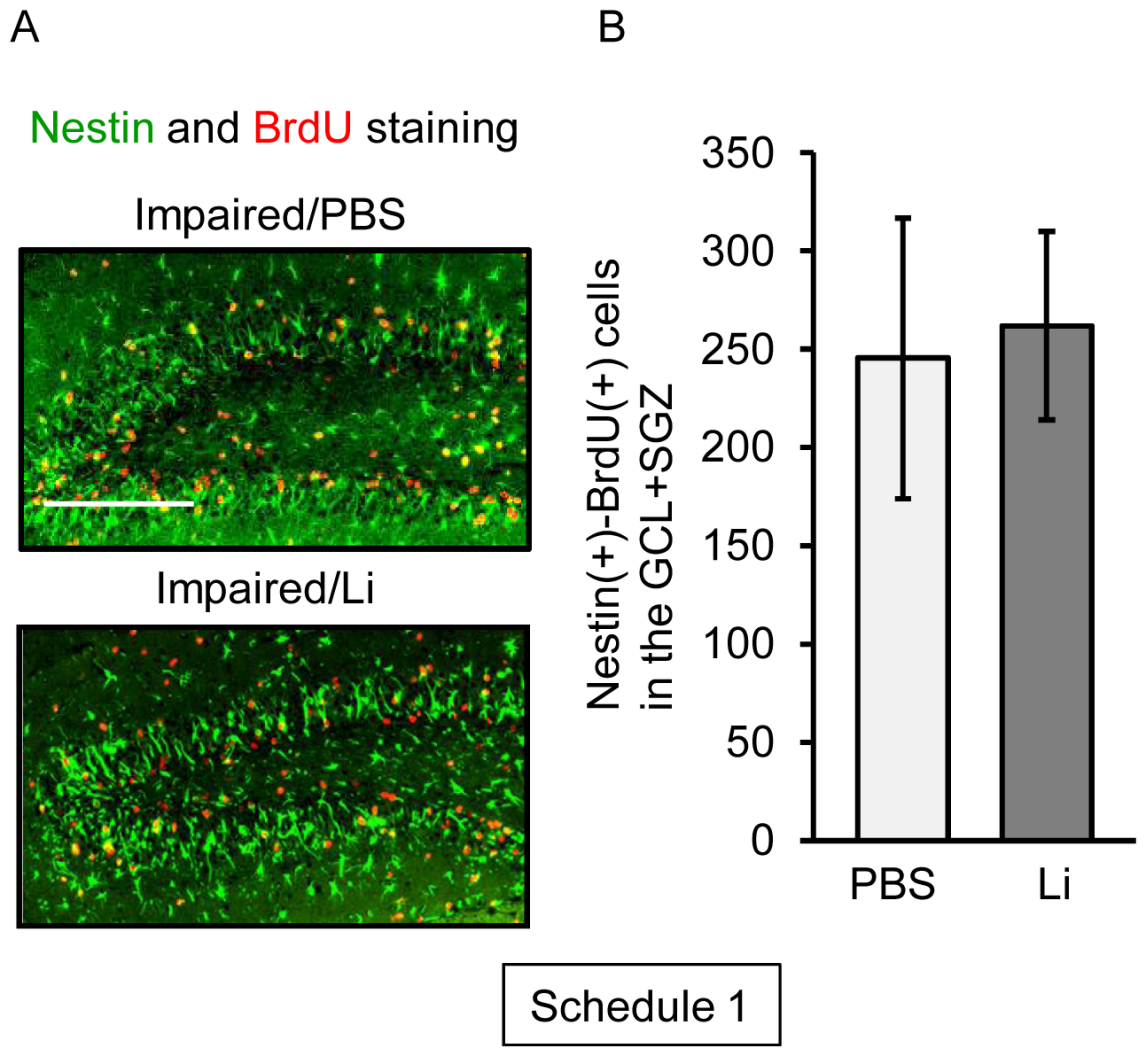
Effect of lithium (Li) on proliferation of nestin(+) cells following neuronal loss. Animals were given either lithium carbonate (100 mg/kg, i.p.) or PBS alone with BrdU on day 2 post-treatment with TMT, and then decapitated on day 3 post-treatment for preparation of sagittal hippocampal sections, which were then stained with antibodies against nestin and BrdU (Schedule 1). (**a**) Fluorescence micrographs show nestin(+) cells (*green*) and BrdU(+) cells (*red*) in the dentate gyrus of the 2 groups (impaired/PBS, impaired/Li). Scale bar = 100 µm (**b**) Graph denoting the number of nestin(+)-BrdU(+) cells in the GCL+SGZ of each group. Values are expressed as the mean ± S.E., calculated from 5 animals.

To assess the effect of the acute treatment with lithium on the generation of neural stem/progenitor cells in the dentate gyrus of the impaired animals, we next determined the number of BrdU(+)-nestin(+) cells in the dentate gyrus on day 3 post-TMT treatment (Figure 3). As found previously [14], [16], the impaired animals had a large increase in the number of nestin(+) cells in their dentate gyrus, mainly in the GCL+SVZ, at the initial time window following the dentate neuronal loss. As expected, lithium was ineffective in changing the number of BrdU(+)-nestin(+) cells in the GCL+SGZ.

### Effect of Lithium on Survival of BrdU(+) Cells Generated following Neuronal Loss in the Dentate Gyrus

**Figure 4:**
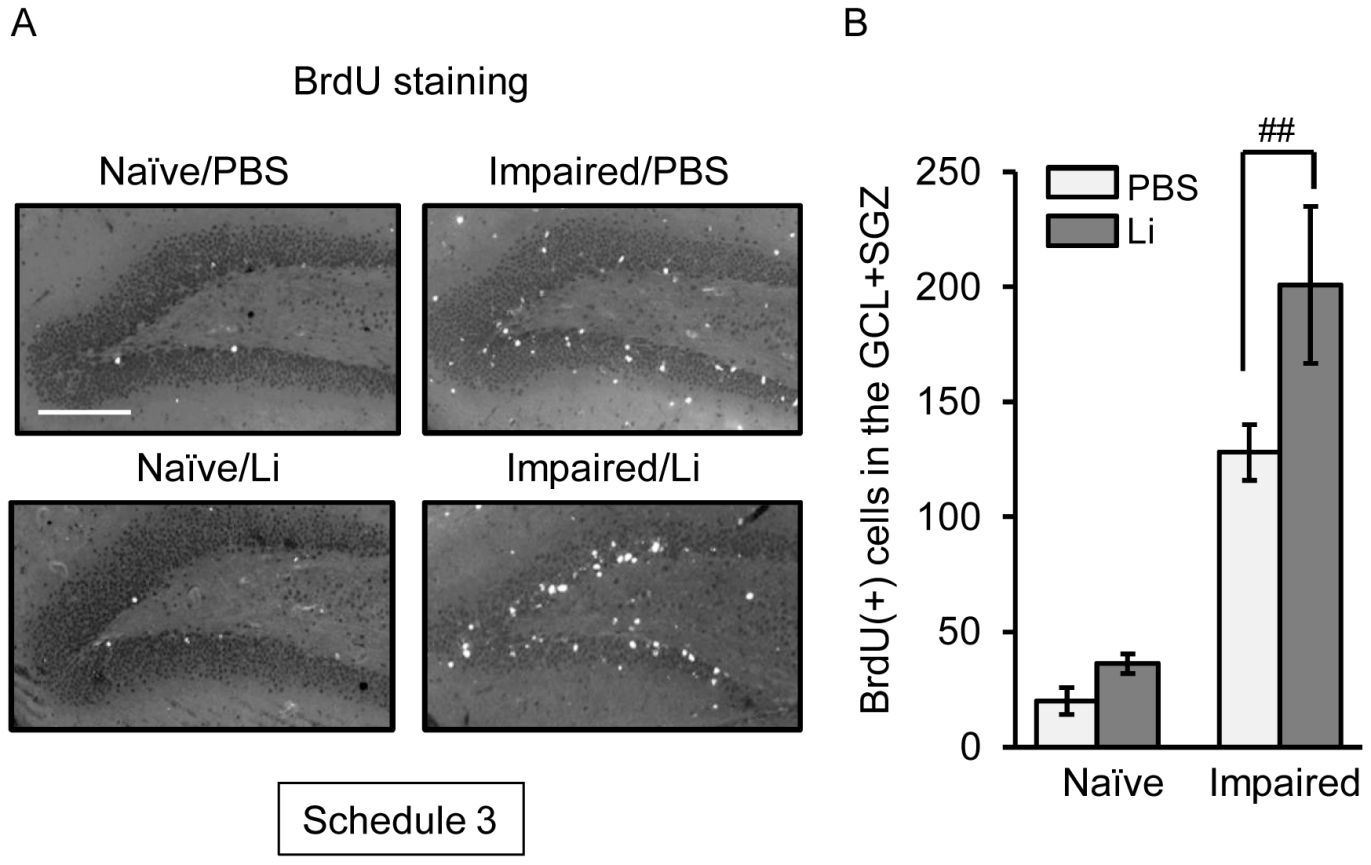
Effect of lithium (Li) on the survival of BrdU(+) cells generated following neuronal loss. Animals were given either lithium carbonate (100 mg/kg, i.p.) or PBS with BrdU on day 2 post-treatment with PBS or TMT, subsequently given either lithium carbonate or PBS up to day 15, and then decapitated on day 30 post-treatment for preparation of sagittal hippocampal sections, which were then stained with anti-BrdU antibody (Schedule 3). (**a**) Fluorescence micrographs show BrdU(+) cells in the dentate gyrus of the 4 groups (naïve/PBS, naïve/Li, impaired/PBS, impaired/Li). Scale bar = 100 µm (**b**) Graph showing the number of BrdU(+) cells in the GCL+SGZ of the 4 groups. Values are expressed as the mean ± S.E., calculated from 5 animals. ^##^ *P*<0.01, significant difference between the values obtained for PBS and Li groups.

Enhanced survival of newly-generated neural progenitor cells is critical for neuronal regeneration following neuronal degeneration. Based on this view point, we next examined the effect of the chronic treatment with lithium on the survival of BrdU(+) cells in the dentate gyrus of naïve and impaired animals. The cell survivability was assessed by counting the BrdU(+) cells remaining in the dentate gyrus on day 30 post-treatment with PBS or TMT (Figure 4). At this time window, the number of surviving BrdU(+) cells in the GCL+SGZ of the impaired animals was larger compared with that in the same region of the naïve ones. As expected, treatment with lithium for 15 days significantly increased the number of BrdU(+) cells in the GCL+SGZ of the impaired animals, but not that in these cell layers of the naïve ones. The number of the BrdU(+) cells in the impaired animals was higher in either of the lithium groups than in the PBS ones. However, the molecular layer and hilus showed no significant change in the number of surviving BrdU(+) cells between the 2 groups.

### Effect of Lithium on Differentiation of BrdU(+) Cells Generated following Neuronal Loss in the Dentate Gyrus

**Figure 5:**
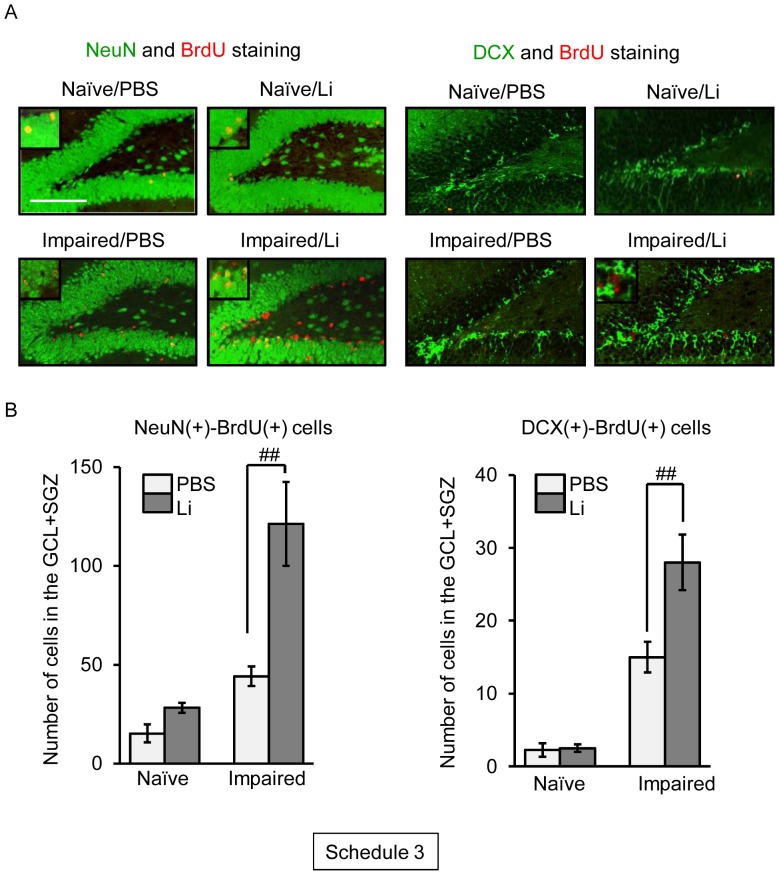
Effect of lithium (Li) on neuronal differentiation of BrdU(+) cells generated following neuronal loss. Animals were given either lithium carbonate (100 mg/kg, i.p.) or PBS with BrdU on day 2 post-treatment with PBS or TMT, subsequently given once a day either lithium carbonate or PBS up to day 15, and then decapitated on day 30 post-treatment for preparation of sagittal hippocampal sections, which were then stained with antibodies against NeuN or DCX and BrdU (Schedule 3). (**a**) Fluorescence micrographs show NeuN(+) cells (*green*) and BrdU(+) cells (*red*) in the dentate gyrus of the 4 groups (naïve/PBS, naïve/Li, impaired/PBS, impaired/Li). Scale bar = 100 µm (**b**) Graphs showing the numbers of NeuN(+)-BrdU(+) cells and DCX(+)-BrdU(+) cells in the GCL+SGZ of the 4 groups. Values are expressed as the mean ± S.E., calculated from 4–11 animals. ^##^ *P*<0.01, significant difference between the values obtained for PBS and Li groups.

To assess the fate of the newly-generated cells in the dentate gyrus following neuronal loss, we carried out double-labeling of BrdU and some neural markers, such as NeuN (mature neurons), DCX (immature neurons), GFAP (astrocytes), and Iba1 (microglial cells), on day 30 post-treatment with PBS or TMT (Figure 5).

Comparing cells positive for both NeuN and BrdU between the naïve and impaired animals, no significant change in the numbers of those cells was observed in the GCL+SGZ. The chronic treatment with lithium increased the number of NeuN(+)-BrdU(+) cells in this region of the impaired animals. However, lithium was ineffective in changing the number of these cells in the GCL+SGZ of the naïve animals. There was also a lithium-induced increase in the number of DCX(+)-BrdU(+) cells seen in the GCL+SGZ of the impaired animals.

**Figure 6:**
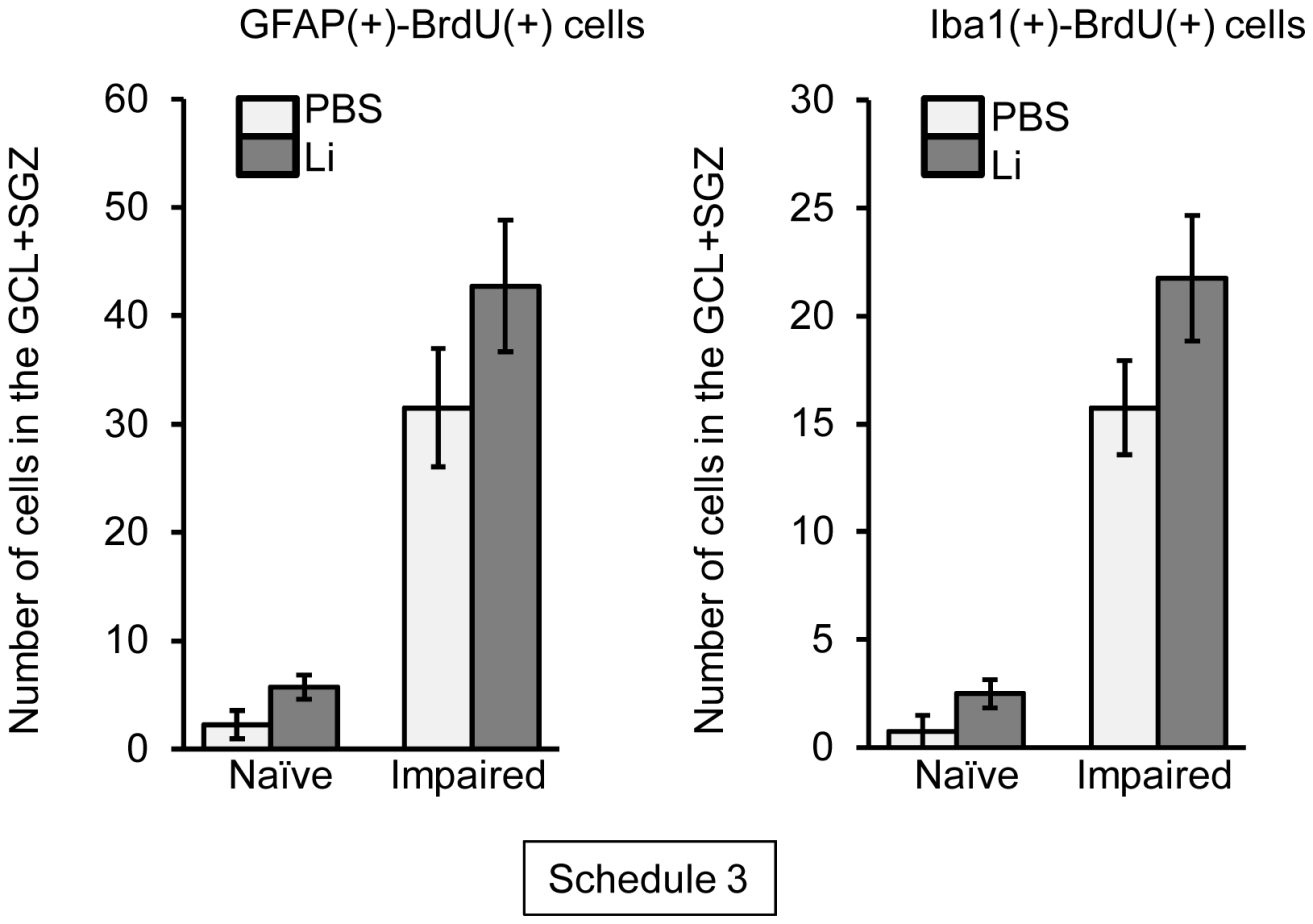
Effect of lithium (Li) on glial differentiation of BrdU(+) cells generated following neuronal loss. Animals were given either lithium carbonate (100 mg/kg, i.p.) or PBS with BrdU on day 2 post-treatment with PBS or TMT, subsequently given once a day either lithium carbonate or PBS up to day 15, and then decapitated on day 30 post-treatment for preparation of sagittal hippocampal sections, which were then stained with antibodies against GFAP or Iba1 and BrdU (Schedule 3). The graphs denote the number of double-positive cells in the GCL+SGZ of the 4 groups. Values are expressed as the mean ± S.E. calculated from 4 animals.

To detect newly-generated astrocytes and microglial cells following neuronal loss in the dentate gyrus of the naïve and impaired animals, we determined the numbers of GFAP(+)-BrdU(+) and Iba1(+)-BrdU(+) cells (Figure 6). GFAP(+)-BrdU(+) cells were not significantly changed in number in the GCL+SGZ between the lithium and PBS groups in either naïve or impaired animals. Similarly, the number of Iba1(+)-BrdU(+) cells in the dentate gyrus was not changed by the lithium treatment.

### Effect of Treatment with Lithium on Nuclear Translocation of β-catenin in BrdU(+) Cells Generated following Neuronal Loss in the Dentate Gyrus

**Figure 7:**
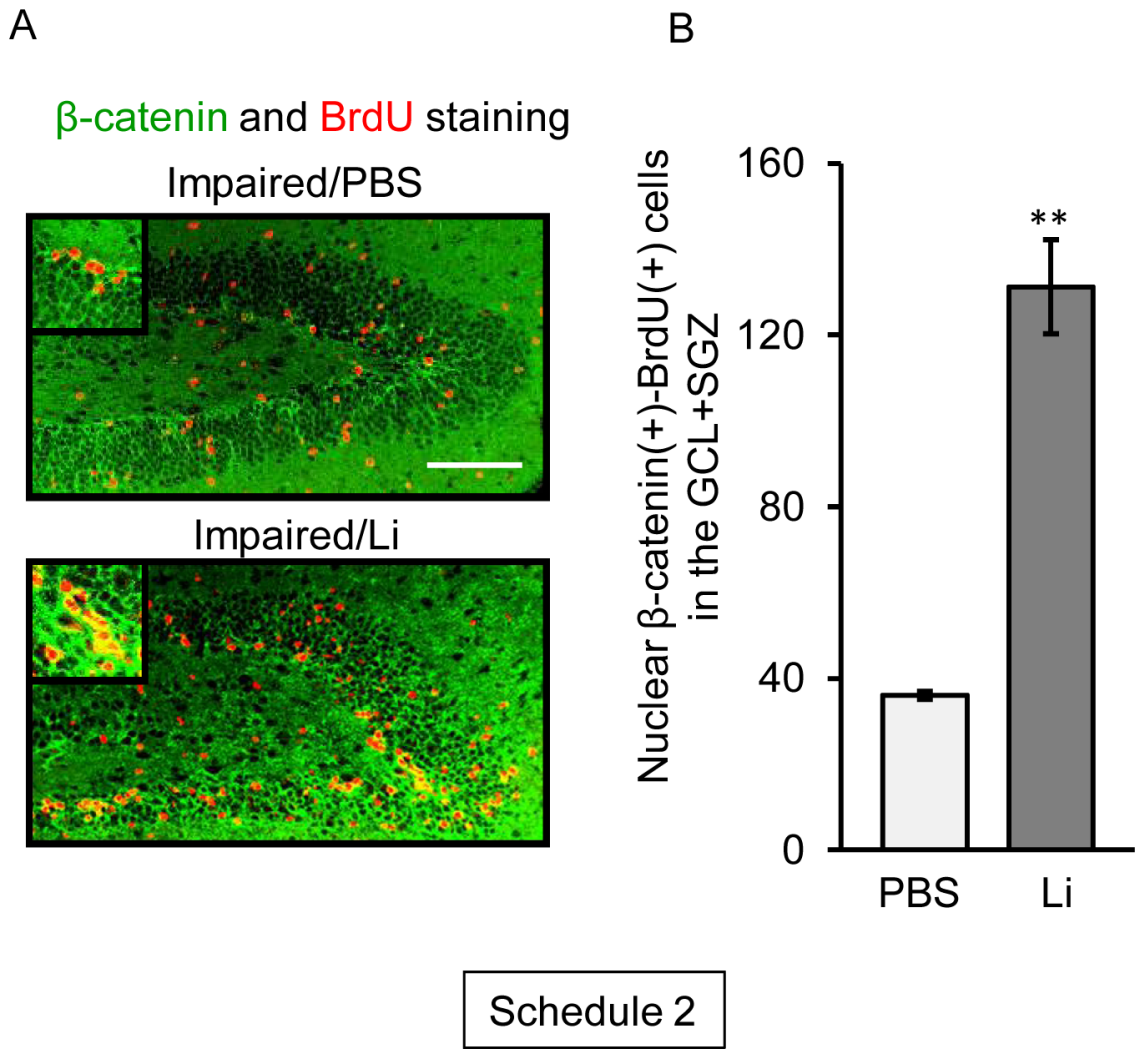
Lithium (Li)-induced nuclear translocation of β-catenin in BrdU(+) cells generated following neuronal loss. Animals were given either lithium carbonate (100 mg/kg, i.p.) or PBS with BrdU on day 2 post-treatment with TMT, subsequently given once a day either lithium carbonate or PBS on days 3 and 5, and then decapitated on day 30 post-treatment for preparation of sagittal hippocampal sections, which were then stained with antibodies against β-catenin and BrdU (Schedule 2). (**a**) Fluorescence micrographs show localization of BrdU (*red*) and β-catenin (*green*) in the dentate gyrus of the 2 groups (impaired/PBS, impaired/Li_2_CO_3_). Scale bar = 100 µm (**b**) Graph denoting the number of BrdU(+) cells with nuclear β-catenin in the GCL+SGZ of each group. Values are expressed as the mean ± S.E., calculated from 5 animals. **P<0.01, significant difference between the values obtained for PBS and Li groups.

The β-catenin/TCF pathway is well known as the canonical Wnt pathway, which regulates the proliferation of embryo-derived NPCs *in vitro* [22] and adult hippocampal neurogenesis *in vivo* [23]. Lithium is an inhibitor of glycogen synthase kinase-3β [24], [25], which is a key regulator of the β-catenin/TCF pathway [26], [27]. Therefore, we examined the effect of lithium on the nuclear translocation of β-catenin in BrdU(+) cells on day 5 post-TMT treatment (Figure 7), when the number of BrdU(+) cells had increased in the GCL+SGZ (Figure 2). Lithium was effective in markedly increasing the nuclear translocation of β-catenin in the BrdU(+) cells in the GCL+SGZ. The ratio of nuclear β-catenin(+)-BrdU(+) cells to total BrdU(+) cells in the GLC+SGZ was also increased by the 3-day lithium treatment on day 5 post-TMT treatment [PBS, 1.6±0.1; Lithium, 2.5±0.2 (P<0.05)].

### Effect of Chronic Treatment with Lithium on Depression-like Behavior following Neuronal Loss in the Dentate Gyrus

Our previous reports demonstrated that following systemic treatment with TMT at the dose of 2.8 mg/kg, approx. 70% of the mice showed “systemic tremor” at 24 h, with this tremor being sustained up to day 3 after the treatment. The remaining (approx. 30%) animals developed “severe tremor” with “motor paralysis in hind limbs.” All TMT-treated mice showed “aggressive” behavior during handling. However, the above behavioral changes elicited by TMT disappeared on day 4 after the TMT treatment [10], [11], [28]. In addition to these behavior abnormalities, impairment of visual recognition memory was observed on day 4 post-treatment with TMT and was ameliorated by day 14 and afterward [14].

**Figure 8:**
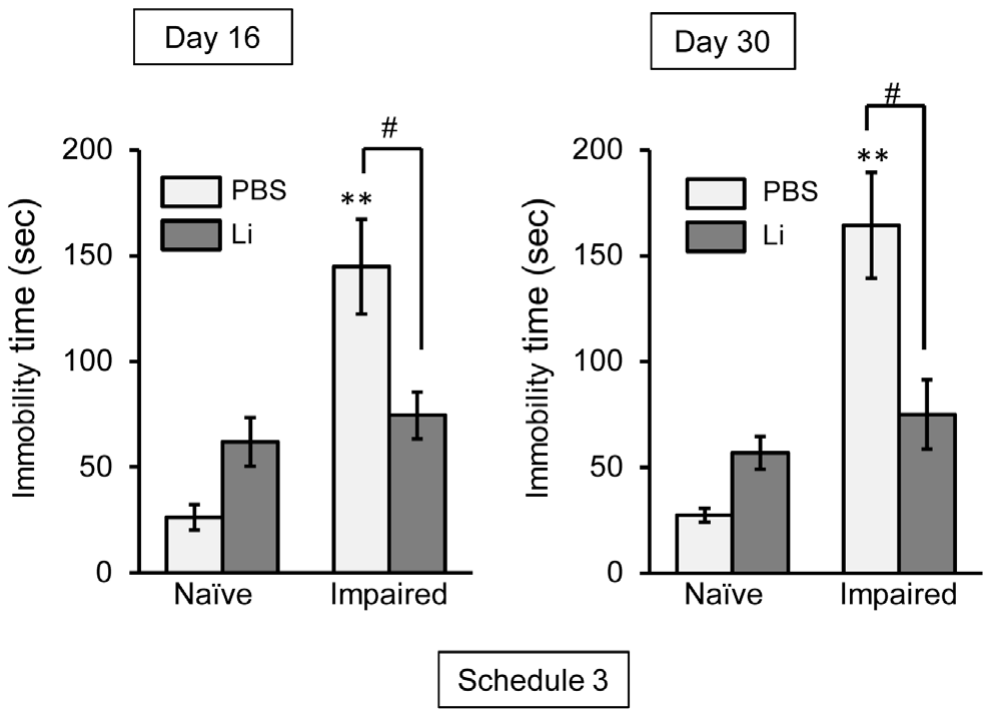
Lithium (Li) ameliorates TMT-induced depression-like behavior. Animals were given either lithium carbonate (100 mg/kg, i.p.) or PBS daily on days 2 to 15 post-treatment with TMT or PBS prior to the forced swimming test, which was conducted on days 16 and 30 post-TMT treatment (Schedule 3). Values are expressed as the mean ± S.E. calculated from 4–6 animals. **P<0.01, significantly different from the control value obtained for the naïve group. ^#^ *P*<0.05, significant difference between the values obtained for PBS and Li groups.

As another abnormal behavior, we focused on delayed depression-like behavior in the impaired animals. In the forced swimming test, immobility time in the PBS-treated mice was markedly prolonged on both days 16 and 30 post-TMT treatment (Figure 8). At the same time windows, the prolonged immobility time in the impaired animals was significantly ameliorated by the chronic treatment with lithium (Figure 8). No significant change in the locomotor activity was observed under any experimental conditions (data not shown).

## Discussion

The important finding stemming from the present study is that lithium had a beneficial effect on neuronal repair through enhanced neurogenesis following neuronal loss in the hippocampal dentate gyrus. Accumulating evidence suggests that NPCs increase in number around the damaged cerebral cortex following cryoinjury [29], ablation injury [30] or controlled cortical impact [31]. In the current study, we used the TMT-treated mouse (impaired animal) as a model for neuronal loss/self-repair in the dentate gyrus. This model shows neuronal loss predominantly in the GCL on day 2 post-TMT treatment (degeneration stage, day 0 to 2 post-TMT treatment), with neurogenesis occurring in the dentate gyrus to repair the GCL after the neuronal loss there [14]. In the histological assessment using this model, we demonstrated that BrdU-incorporating cells positive for nestin or DCX were dramatically increased in number in the dentate gyrus at the repair stage. The finding that cells positive for both BrdU and NeuN were also observed in the dentate GCL on day 30 post-TMT treatment suggests that the cells newly-generated following neuronal loss in the GCL had the ability to differentiate into neuronal cells. Behavioral assessment in this model reveals that cognition impairment is observed in the mice during the degeneration stage, with recovery at the repair stage [14], [28]. However, the current data showing that the depression-like behavior was observable in the PBS group even on day 30 post-TMT treatment allows us to propose that neuronal repair in the hippocampus of TMT-treated mice is incomplete under the condition without lithium treatment. Importantly, the present data showed that the chronic treatment with lithium ameliorated the depression-like behavior in this model, suggesting that lithium was effective in facilitating functional neuronal repair after neuronal loss in the dentate gyrus.

The neurogenesis process in adults is accomplished by at least 3 steps including the proliferation, migration, and survival/differentiation of NPCs. For elucidating the effect of lithium on the neurogenesis process, we used 3 types of experimental schedules. One was a single treatment with lithium performed simultaneously with the first injection of BrdU on day 2 post-TMT treatment in order to evaluate the effect of lithium on the proliferation of NPCs [BrdU(+)-nestin(+) cells] following neuronal loss in the dentate gyrus (Schedule 1). As the acute treatment with lithium had no effect on the expression of BrdU-incorporating cells under the present experimental conditions, we gave lithium daily on days 2 to 4 post-TMT treatment (Schedule 2). To address the fate (survival/differentiation) of the newly-generated cells on day 30 following neuronal loss in the dentate gyrus, we evaluated the effect of the chronic (13 days) treatment with lithium on the BrdU-incorporating cells positive for NeuN, DCX, Iba1, and GFAP (Schedule 3). In addition to the behavioral assessment, the current data under experimental Schedule 3 showed that the chronic treatment with lithium had a beneficial effect on the neuronal repair in this animal model.

Accumulating evidence suggests that 4 different cell populations (type 1, 2a, 2b, and 3 cells) in the dentate gyrus are involved in the adult neurogenesis process [32]–[34]. The type 1 cell is classified as a radial glia-like cell located in the SGZ. These cells cross into the GCL and rarely enter into the cell cycle (slow-cycling cell). The type 2a cell is the amplifying progenitor, which is located in the SGZ and enters into the cell cycle more often (fast-cycling cell). These cells are proposed to be derived from type 1. The type 3 cell is a neuroblast without proliferative activity, and it differentiates into a mature neuron that migrates into the GCL. *Ex vivo* findings obtained on cells prepared from the dentate gyrus of naïve and impaired mice suggest that the population of type 1 [nestin(+)-GFAP(+) cell] is about 3-fold greater in number than that of the type 2a [nestin(+)-GFAP(−) cell] in naïve animals, whereas the type 2a population is about 1.5-fold greater than that of type 1 at the initial time window of the repair stage in the impaired animals [16]. These findings suggest that at the initial time window of the repair stage in impaired animals, the type 2a cell was greater in number than the type 1 cell, although both type 1 and type 2a cells were increased in number in the dentate gyrus. Under experimental Schedule 2, the data showing that 3-day treatment with lithium increased the number of BrdU(+) cells in the GCL+SGZ might be evidence for promoted proliferation of type 1 and 2a cells at the initial time window of the repair stage following neuronal loss in the dentate gyrus. However, the single treatment with lithium was ineffective in increasing BrdU incorporation in the GCL+SGZ on day 3 post-TMT treatment. This finding might indicate that lithium had no immediate effect on proliferation of NPCs in the hippocampus.

Lithium is an inhibitor of glycogen synthase kinase-3β [24], [25], which is widely known as a key regulator of the β-catenin/TCF pathway [26], [27]. The activation of this pathway is known to increase cyclin D1 expression in tumor-derived cell lines [35], [36]. It has been shown that the β-Catenin/TCF pathway is the canonical Wnt pathway, which regulates the proliferation of embryo-derived NPCs *in vitro* [22] and adult hippocampal neurogenesis *in vivo* [23]. The Wnt pathway regulates the proliferation of NPCs in the late stages of differentiation [37], as well as in the early differentiation stage [20]. In the present study, we showed that lithium treatment increased the number of newly-generated cells with a high level of nuclear β-catenin at the initial time window (5 day post-TMT treatment) of the self-repair stage. Therefore, these suggest that lithium enhanced the proliferation of NPCs in the early differentiation stage through activation of the β-catenin/TCF pathway in the hippocampal dentate gyrus. Furthermore, Boku et al. [20] demonstrated that lithium recovers dexamethasone-induced decrease in NPC proliferation in the dentate gyrus, but not in naïve dentate gyrus. This previous report and our current data support the idea that lithium has the ability to promote the recovery of the impaired dentate gyrus through enhanced the proliferation of NPCs during hippocampal neurogenesis.

In the present study, we found a dramatic increase in the number of BrdU(+)-NeuN(+) cells and BrdU(+)-DCX(+) cells in the GCL on day 30 post-TMT treatment by chronic treatment with lithium. However, the number of BrdU(+)-GFAP(+) cells (astrocytes) or BrdU(+)-Iba1(+) cells (microglial cells) was not affected by lithium under the same conditions. Importantly, newly-generated neuronal cells [BrdU(+)-NeuN(+) and BrdU(+)-DCX(+) cells] were located predominantly in the GCL. These data suggest that lithium was capable of differentiating newly-generated cells into neuronal cells, which then migrated to the dentate GCL. The finding that lithium had no significant effect on the newly-generated neuronal cells in the GCL of naïve animals indicates that the lithium-induced enhancement of hippocampal neurogenesis was selective in affecting only the impaired dentate gyrus. In agreement with the above findings, the TMT-induced depression-like behavior was improved by lithium. It is most likely that the enhanced hippocampal neurogenesis following neuronal impairment of the dentate gyrus is regulated by mechanisms different from those underlying that in the intact dentate gyrus. This interesting possibility can and should be evaluated by using the present model for neuronal loss/self-repair in the dentate gyrus.

## Conclusion

We provided, for the first time, evidence for the ability of lithium to promote NPC proliferation and survival/neuronal differentiation of newly-generated cells in the dentate gyrus following neuronal loss caused by *in vivo* treatment with TMT. Hence, it is possible that lithium is capable of facilitating neurogenesis after neuronal damage in the dentate gyrus of adult animals. The goal is the development of new regenerative medical techniques for the treatment of brain insults.
